# Influence of ethanolic extract of *Allium sativum* on *TP53* gene and its anticancer potential in N-Nitrosodiethylamine (NDEA)-induced hepatocellular carcinoma in male albino rats

**DOI:** 10.22038/IJBMS.2022.62295.13787

**Published:** 2022-04

**Authors:** Godwin Offumobi Ogar, Joseph Bamidele Minari, Adebayo Joseph Bello, Janet Chiwetalu, Oluwafunto Eunice Omogunwa, Oluwadamilola Suzan Oshikoya, Micheal Tobiloba Otaru, Chioma Anastacia Anyanele

**Affiliations:** 1 Cancer Research and Infectious Disease Unit, Department of Cell Biology and Genetics, Faculty of Science, University of Lagos, Akoka, Lagos

**Keywords:** Antineoplastic agents, Carcinogens, Carcinoma, Diethylnitrosamine, Garlic, Hepatocellular, Medicinal, p53

## Abstract

**Objective(s):**

Cancer is a group of genetic disorders in which the behavior of the cell is disturbed by mutation and other abnormalities thereby posing as the leading cause of morbidity and mortality globally. Hepatocellular Carcinoma (HCC) is the most common form of liver cancer, highly aggressive with high mortality and incidence rate; and has limited therapeutic options. Most of the conventional cancer chemotherapeutics are associated with undesirable side effects, toxicity, chemoresistance, and high treatment cost, driving the need for a safer and more effective treatment alternative. Medicinal plants and herbs have shown very promising anti-cancer properties which are important for cancer treatment due to their multiple chemical compounds.

**Materials and Methods:**

Qualitative screening of the ethanolic extractof *Allium sativum *was conducted showing the different phytochemicalspresent. The levels of liver function and hematological parameters wasdetermined via spectrophotometric analysis. Polymerase Chain Reaction techniquewas used to assess the gene patterns of Tumorsuppressor p53 (TP53).

**Results:**

Phytochemical analysis revealed that *Allium sativum* has properties that antagonize the proliferating process of carcinogenesis in the liver. The NDEA-group showed significant distortion in the liver architecture characterized by vascular congestion of blood sinusoids, cirrhosis, and congestive hepatopathy while the treated groups showed a reduction in the abnormalities and malignant formation. The treated group showed a significant (*P*<0.05) increase and restored activities of Alanine aminotransferase (ALT), Aspartate aminotransferase (AST), Alkaline phosphatase (ALP), Bilirubin and hematological parameters (RBCs, WBCs, and Platelets). *TP53* gene amplification was significantly (*P*<0.05) visible after treatment.

**Conclusion:**

Ethanolic plant extract of *A. sativum* demonstrates its anticancer properties by improving the liver architecture, increasing the antioxidant defense systems, and activation of the tumor suppressor (*TP53*) gene. Garlic extract has anti-proliferating properties and can be used as an alternative mode of treatment and prevention for hepatocellular carcinoma.

## Introduction

Cancer is currently being ranked as the leading cause of death globally due to daily increase in the number of deaths (1). Liver cancer is the sixth most commonly diagnosed cancer which remains a global health challenge, with an estimated incidence of >1 million cases by 2025 and the third leading cause of cancer death worldwide in 2020, with approximately 906,000 new cases and 830,000 deaths (2, 3). According to Global Cancer Observatory 2021, Nigeria recorded 5180 new cases of liver cancer and 5046 deaths (4). Primary liver cancer includes hepatocellular carcinoma (HCC) which is very common (comprising 75%–90% of cases) and intrahepatic cholangiocarcinoma (10%–15%), as well as other rare types (2, 3). Chemotherapy and radiotherapy are mainstay treatments for cancer patients. However, their clinical outcomes are highly limited by the resistance of malignant tumors to these therapies and the incurrence of serious damages to vital organs (5).

HCC is known to be a highly aggressive cancer with mortality running parallel to its incidence and prevalence which limits its therapeutic options. Chronic infection with hepatitis B virus (HBV) or hepatitis C virus (HCV), carcinogens (tobacco smoking, food contaminants, and environmental toxins), and inherited diseases are the major risk factors of hepatocellular carcinoma. Several factors such as gender, age, ethnicity, and demographic regions also increase the HCC incidence rate (6).

Tumor suppressor p53 (*TP53) *is considered the “Guardian of the genome” to prevent the accumulation of oncogenic mutations that lead to a malignant tumor. Tumor suppressor p53 (*TP53)* is the most frequently mutated tumor suppressor gene in human cancer and also the principal cellular responder to various stress signals such as DNA damage, oncogene activation, hypoxia as well as reactive oxygen species (ROS). The activation of p53 induces numerous cellular responses such as cell cycle arrest to restore genetic integrity, or programmed cell death (apoptosis), senescence, or ferroptosis to eliminate unrecoverable cells. (7–9).

The use of medicinal plants for the management of cancer has been in continuous use over the years, and has received enormous attention, particularly, in most developing countries of the world such as Nigeria. The treatment of cancer using synthetic chemical drugs is often accompanied by harsh, toxic, and deleterious side effects. Thus, the alternative use of readily available and inexpensive medicinal plants is the panacea to the toxic side effects associated with synthetic drugs. (1); although their use belongs to the traditional treatment regimes, plant-derived compounds still cover a large portion of the current-day pharmaceutical agents. Their medical importance is well recognized in the field of oncology, especially as an alternative to the limitations of conventional chemotherapy (severe side effects and inefficacy due to the occurrence of multi-drug resistance) (10). Garlic (*Allium sativum*) is among the oldest of all cultivated plants. It has been used as a medicinal agent for thousands of years. Garlic is a remarkable plant that has been used as a medicinal herb due to its therapeutic activities and multiple beneficial effects such as antimicrobial, antithrombotic, hypolipidemic, antiarthritic, hypoglycemic, and antitumor activity (11–14).

N-nitrosodiethylamine (NDEA) is considered one of the most common environmental chemicals that are known to be metabolized to carcinogenic and pro-oxidant agents. It usually exists in processed meats, tobacco products, alcoholic beverages, and agricultural chemicals (15). The carcinogenic effect of NDEA is associated with a highly generated ROS which could damage bio-molecules such as DNA, proteins, and lipids. NDEA could cause the formation of large amounts of 8-hydroxy-2-deoxyguanosine at a very low dose in the liver of rats, which could then initiate carcinogenesis. Hepatocarcinogenesis induced by NDEA is a well-known animal model commonly used for the screening of the hepato-protective activity of natural compounds (16). 

This study aims to evaluate the anticancer effect of ethanolic extract of *A. sativum* on liver cancer induced by Nitrosodiethylamine (NDEA) in albino rats.

## Materials and Methods

***Collection and identification of plants***

A sample of fresh cloves of garlic (*A. sativum)* was purchased from a local market in Sabo-Yaba located in Lagos state in the month of February 2021. The plant was identified in the herbarium unit of Botany Department, University of Lagos, and authenticated by a taxonomist with the voucher specimen number 8815.

***Preparation of ethanolic extract of A. sativum***

The ethanolic extract of *A. sativum* was obtained using the modified conventional method described by Gonelimali *et al*. (17).  10 g powder of the plant material (*A. sativum* bulb) was mixed with ethanol (9:1) separately in round bottom flasks and incubated at 37 °C and 150 rpm overnight. Liquid extracts obtained were separated from the solid residue by filtration using Whatman No. 1 filter, and then concentrated using a rotary evaporator at 40 °C to give a final weight of 161.8 g of the extract and percentage yield of 3.6.

***Chemical***

Nitrosodiethylamine (NDEA) was purchased from Bristol scientific company (UK). All other chemicals were obtained commercially and are of analytical grade.

***Animals***

 All experimental albino rats (40 male albino rats were used with an average weight of 130 g) were purchased from the Department of Zoology, University of Lagos with specimen identification number 10112. The animals were bred in the laboratory under ideal conditions of temperature, humidity, and light. The work was carried out in the animal house (Botanical garden), University of Lagos in accordance with the Code of Ethics of the World Medical Association for animal experiments. Approval was obtained from the University of Lagos Ethics Committee for conformity to Guidelines for Experiments with Whole Animals.

***Experimental design and tumor induction***

 The rats** (**40) with an average weight of 130 g were divided into 8 treatment groups. Six groups (A to F) were induced intraperitoneally (IP) with 100 mg/kg of NDEA (10 ul diluted to 1 ml with 0.15M sterile NaCl) and treated with daily doses of (50, 100, 200, 300, 400, and 500) mg/kg body weight of ethanolic extract of *A. sativum* for a period of seven weeks; while the Positive Control (PC) experiment received feed with distilled water and the Negative Control (NC) was induced with NDEA only as follows:

Group PC (Positive Control): Control experiment fed with distilled water and food only

Group NC (Negative Control): NDEA-induced rats only

Group A: NDEA-induced rats treated with ethanolic extract from *A. sativum* (50 mg/Kg)

Group B: NDEA-induced rats treated with ethanolic extract from *A. sativum* (100 mg/Kg)

Group C: NDEA-induced rats treated with ethanolic extract from *A. sativum* (200 mg/Kg)

Group D: NDEA-induced rats treated with ethanolic extract from *A. sativum* (300 mg/Kg)

Group E: NDEA-induced rats treated with ethanolic extract from *A. sativum* (400 mg/Kg)

Group F: NDEA-induced rats treated with ethanolic extract from *A. sativum* (500 mg/Kg)

***Phytochemical screening of ethanolic extract from A. sativum***

The qualitative phytochemical properties of *A. sativum *plant extracts were determined according to the methods of Sofowora, (18) and Trease and Evans (19). 

***Histological determination***

For microscopic evaluation, liver tissues were fixed in a fixative (10% formal saline) and embedded in paraffin, sectioned 4–5µm, and subsequently stained with hematoxylin&eosin. A section was studied under a light microscope at 40 to 100 magnifications. Slides of all the treated groups were studied and photographed.

***Hematology***

Blood was collected at the time of euthanasia by cardiac puncture and was used for hematological and serological analysis. White blood cell (WBC) count, red blood cell (RBC) count, packed cell volume (PCV), and total protein levels of all rats were determined using the standard protocol.

***Liver function test***

The liver function test was carried out to ascertain the effects of *A. sativum* extract on the enzymatic biomarkers (Aspartate aminotransferase (AST) and Alanine aminotransferase (ALT)), Serum glutamic-oxaloacetic transaminase (SGOT), Serum glutamic pyruvic transaminase (SGPT), Serum creatinine, and PHOS (Alkaline phosphatase) produced in the liver and was used in determining the state of the liver of the rats. Blood samples were collected from all groups before the specimen was sacrificed.

***Polymerase chain reaction***

Nucleic acid extraction kit (Zymo Research) was used for DNA isolation from the liver tissues. This was followed by PCR amplification using specific *TP53* primers. The primer sets were: Forward: ACAGCAAGGATACACACAAGAAG Reverse: CCAGCACGGAGTACCAGTA (Location: Exon 9). Other relevant information can be obtained from (https://www.ncbi.nlm.nih.gov/nuccore/NC_051345.1?report=fasta).

The PCR reaction was carried out using the following PCR amplification profile:

Initial denaturation: – 94 °C for 5 min

Denaturation: – 94 °C for 1 min

Annealing: – 55 °C for 1 min

Extension: – 72 °C for 2 min

Final extension: – 72 °C for 7min

Final hold: – 4 °C

PCR was performed in 25 µl final volumes, starting with 2 µl template DNA, 12.5 µl master mix consisting of dNTPs, MgCL_2 _solution and Taq polymerase, 1.5 µl reverse primer, 1.5 µl forward primer, 7.5 µl distilled water. The reaction mixture was heated under the following thermal profile. Initial denaturation at 95 °C for 10 min followed by 40 cycles: denaturation at 94 °C for 30 sec, annealing at 60 °C for 30 sec, extension at 72 °C for 10 min. The assembled reaction was sealed, vortexed, centrifuged, and placed in the thermocycler for DNA amplification. The amplification was carried out in a Techne TC-412 Thermocycler. The PCR amplification products were subjected to electrophoresis (BioRad, USA) in 1% agarose gel in 0.5x concentration of Tris-borate-EDTA buffer at 70V for 1 hr and stained with ethidium bromide; it was then visualized under ultra-violet trans-illuminator and the expected bands were observed using a Polaroid camera. Gel analysis was done using GelAnalyzer 19.1.

***Statistical analysis method***

The data obtained were analyzed using GraphPad version 8 and the results were expressed as Mean ± SEM. Significant differences were established by using one-way ANOVA. A difference was considered significant at *P<*0.05.

## Results

***Phytochemical results***

The qualitative phytochemical analysis results of the garlic extract used for this study are shown in Table 1. The results showed the presence of tannins, phenolics, and reducing sugars; and the absence of phlobatannins, cardiac glycosides, flavonoids, and terpenoids.

***Body weight results***

**Figure 1:**
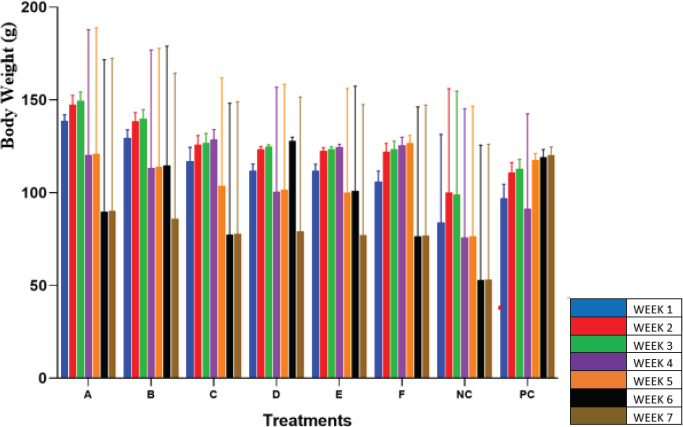
Bodyweight of Nitrosodiethylamine (NDEA) induced rat administered with different concentrations of garlic extract per group per week

The results observed from the analysis of mean body weight showed a significant decrease in the bodyweight of the albino rats treated with a lesser concentration of garlic extract (Figure 1). Results obtained from this study revealed that for the first 3 weeks, there was no significant difference (*P*>0.05) in the mean bodyweight of the experimental animals across all groups compared with the PC group as a control group. In week 4, there was a significant decrease (*P*<0.05) in the mean body weight of groups A, B, D, and NC. Groups A, B, and D showed a significant decrease (*P*<0.05) in the mean body weight from week 4 down to week 7, while groups C and E showed a significant decrease (*P*<0.05) in the mean body weight from week 5 down to week 7; however, Group D showed an increase in the mean body weight in week 6. Group F showed a significant decrease (*P*<0.05) in the mean body weight at week 7 while group NC showed a decrease in the mean body weight only at weeks 4 and 5. It was observed that the higher the concentration of garlic extract given to the test specimen, the lower the decrease in the mean body weight.

***Survival and Death Rate Result***

**Figure 2:**
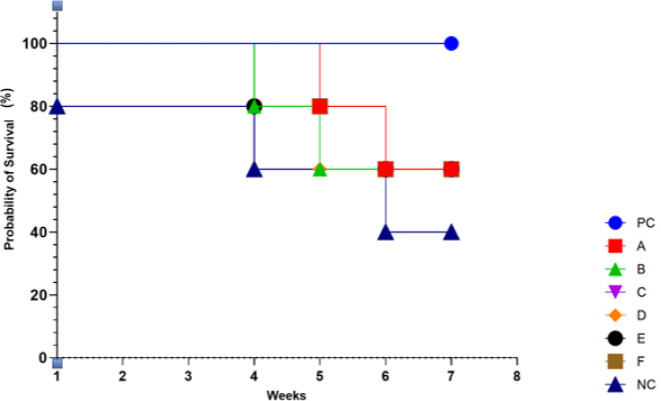
Mortality rate analysis of test specimen treated with different concentrations of garlic extract

Figure 2 shows the mortality rate of the experimental specimen used for this study. The results obtained from this study revealed that no death rate was recorded for group PC. Group NC, started with an 80% survival rate and this declined in week 4 to a 60% probability of survival and which further declined at week 6 to a 40% probability of survival. All other groups started with a 100% probability of survival but a decline to 80% survival rate was observed at week 4 except group A whose decline in survival rate was observed at week 5. All group’s survival rate declined to a 60% probability rate during the 7 week course of induction except for group PC where no decline was recorded and group NC where the decline fell further to a 40% survival rate.

***Histopathology***

**Plate 1:**
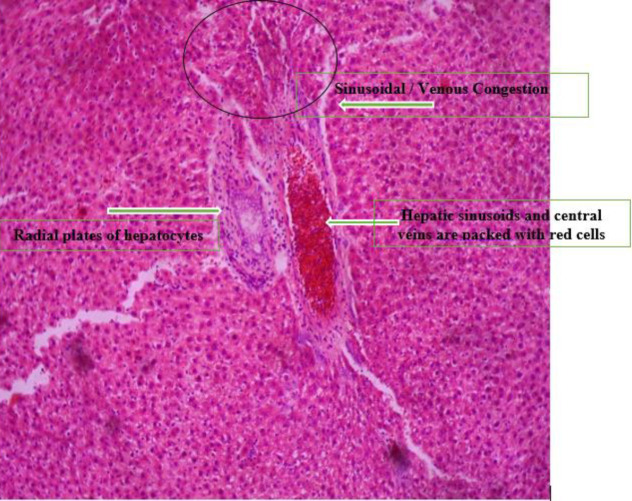
Photomicrograph of Group A liver which shows radial plates of hepatocytes. The hepatic sinusoids and central veins are packed with red cells. Sinusoidal/venous congestion was also observed. Group A: Nitrosodiethylamine (NDEA)-induced rats + ethanolic extract from *Allium sativum* (50 mg/Kg)

Plate 1 shows the photomicrograph of Group A liver which shows radial plates of hepatocytes. The hepatic sinusoids and central veins are packed with red cells. Sinusoidal/venous congestion was also observed. 

**Plate 2:**
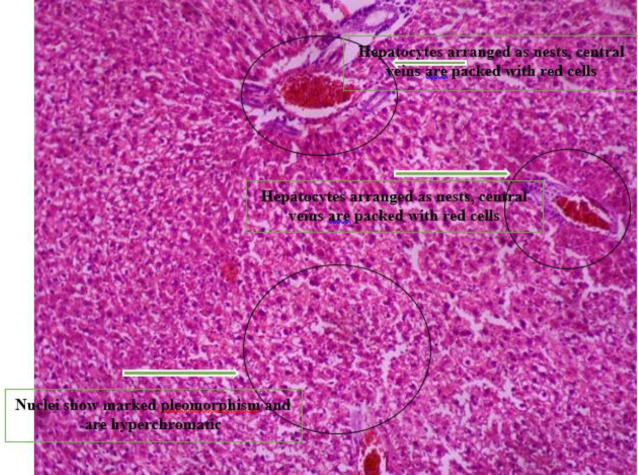
Photomicrograph of Group B liver which shows hepatocytes arranged as nests, central veins are packed with red cells. Their nuclei show marked pleomorphism and are hyperchromatic. Group B: NDEA-induced rats + ethanolic extract from *Allium sativum* (100 mg/Kg)

Plate 2 shows the photomicrograph of group b liver which shows hepatocytes arranged as nests. Their nuclei show marked pleomorphism and are hyperchromatic. 

**Plate 3:**
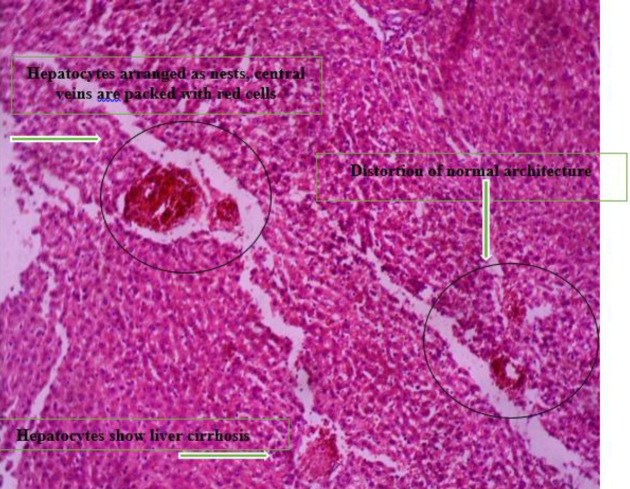
Photomicrograph of group C liver which shows distortion of normal architecture, with formation of islands bounded by fibrous septae. The sinusoids and central veins are packed with red cells. Hepatocytes show liver cirrhosis. Group C: Nitrosodiethylamine (NDEA)-induced rats + ethanolic extract from *Allium sativum* (200 mg/Kg)

Plate 3 shows the photomicrograph of group c liver which shows distortion of normal liver architecture, with formation of islands bounded by fibrous septae. Hepatocytes do not show cytologic atypia but show liver cirrhosis. 

**Plate 4:**
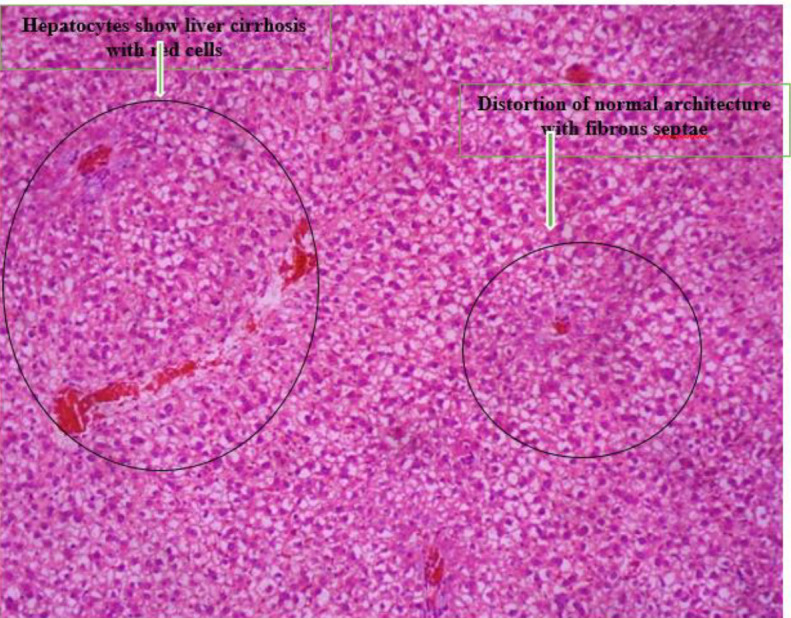
Photomicrograph of Group C liver which shows distortion of normal architecture, with formation of islands bounded by fibrous septae. Hepatocytes show liver cirrhosis. Group D: NDEA-induced rats + ethanolic extract from *Allium sativum* (300 mg/Kg)

Plate 4 shows the photomicrograph of Group C liver which shows distortion of liver normal architecture, with formation of islands bounded by fibrous septae. Hepatocytes show liver cirrhosis. 

**Plate 5:**
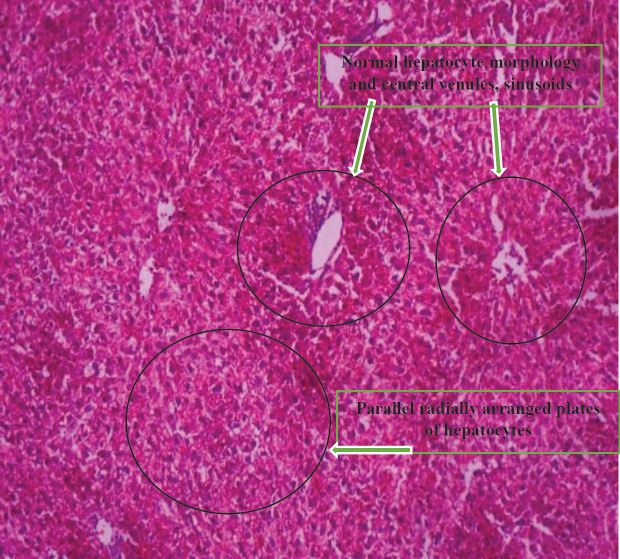
Histologic sections of liver tissue showing parallel radially arranged plates of hepatocytes. Showing normal hepatocyte morphology, normal central venules, and sinusoids. Group E: NDEA-induced rats + ethanolic extract from *Allium sativum* (400 mg/Kg)

Plate 5 shows the histologic sections of liver tissue showing parallel radially arranged plates of hepatocytes. 

**Plate 6:**
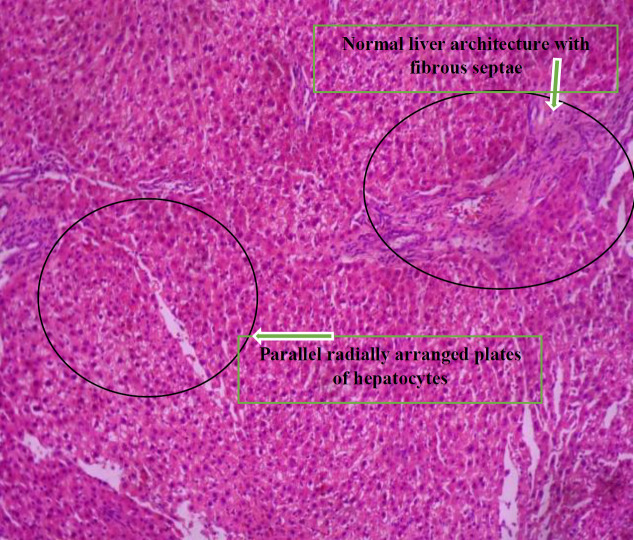
Histologic sections of liver tissue of group F showing parallel radially arranged plates of hepatocytes, normal liver architecture with fibrous septae. Group F: NDEA-induced rats + ethanolic extract from *Allium sativum *(500 mg/Kg)

Plate 6 shows the histologic sections of liver tissue of group F showing parallel radially arranged plates of hepatocytes. 

Plate 8 shows the histologic sections of liver tissue of group G showing normal hepatocyte morphology ,central venules, and sinusoids.

**Plate 7:**
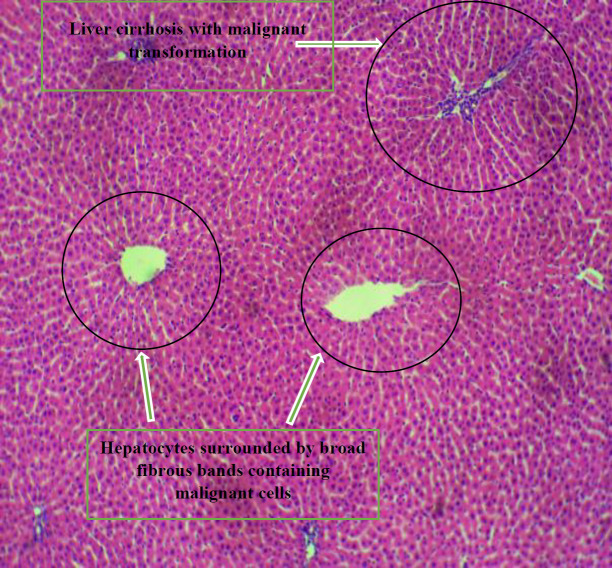
Histologic sections of liver tissue showing islands of hepatocytes surrounded by broad fibrous bands. A focus containing malignant cells is seen. Liver cirrhosis with malignant transformation was observed. Group NC (Negative Control): NDEA-induced rats only

Plate 7 shows the histologic sections of liver tissue showing islands of hepatocytes surrounded by broad fibrous bands. A focus containing malignant cells is seen. Liver cirrhosis with malignant transformation was observed. 

***Liver function test results***

Table 2 shows the effects of ethanolic extract of *Allium sativum* (50 mg/Kg, 100 mg/Kg, 200 mg/Kg, 300 mg/Kg, 400 mg/Kg, and 500 mg/Kg) on N-diethylnitrosamine-induced hepatocellular carcinoma in male albino rats on selected liver enzymes. Values are reported as mean ± SEM of 6 replicates; *P<0.001 is considered significant as compared with negative control; #*P<*0.001 is considered significant as compared with normal control. The highest bilirubin content was observed in the test organism in group A, followed by those in group D. The lowest bilirubin content was found in the experimental animal in group PC.

***Haematology parameter analysis***

**Figure 3:**
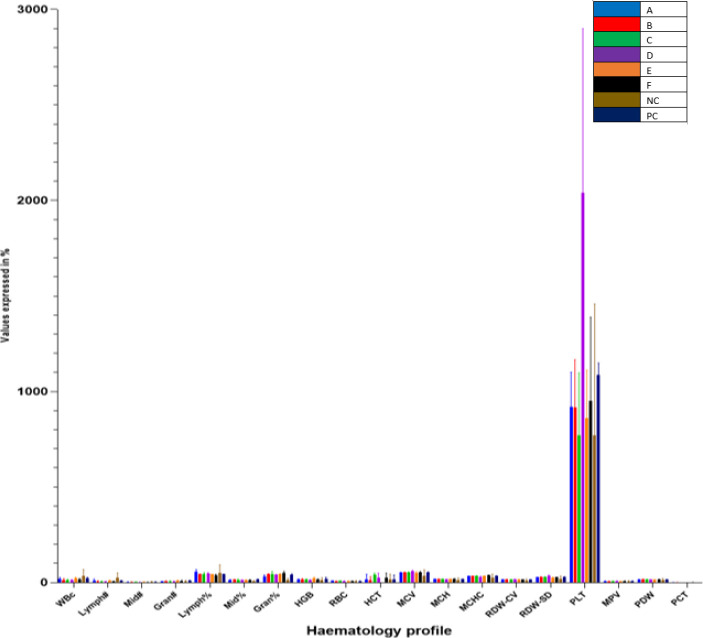
Hematology profile of the rats administered with different concentrations of garlic in NDEA induced liver cancer

Table 3 shows the hematology profile of the NDEA cancer-induced rats while Figure 3 shows the graphical representation of the data. At the end of the administration period, it was observed that the volume of WBC significantly decreased (*P*<0.05) in the induced rats in group D when compared with the positive control. The amount of Red Blood Cell Distribution Width (RDW-CV) in the rats in group F also increased significantly when compared with the positive control group. Procalcitonin (PCT) value in groups D and Negative and Positive control was zero. RBC levels of rats treated with different concentrations of the extract did not change significantly. Generally, it was observed that there are no significant differences in the values of all other hematology profiles of the treated rats across treatment groups when compared with the control.

***Polymerase chain reaction (PCR) results***

The results from the polymerase chain reaction show amplification of the TP53 gene. The amplification of isolated DNA from the blood of control and experimental rats by gel electrophoresis is shown in plate 1. DNA bands in rats receiving varying doses of ethanolic extract of *A. sativum* (50, 100, 200, 300, 400, and 500) mg/Kg are seen in the wells. Amplicons were observed in groups A to G except for the positive control group H. 

Key: Group PC (Positive Control): Control experiment fed with distilled water and food only

Group NC (Negative Control): NDEA-induced rats only

Group A: NDEA-induced rats + ethanolic extract from *Allium sativum *(50 mg/Kg)

Group B: NDEA-induced rats + ethanolic extract from *A. sativum* (100 mg/Kg)

Group C: NDEA-induced rats + ethanolic extract from *A. sativum* (200 mg/Kg)

Group D: NDEA-induced rats + ethanolic extract from *A. sativum* (300 mg/Kg)

Group E: NDEA-induced rats + ethanolic extract from *A. sativum* (400 mg/Kg)

Group F: NDEA-induced rats + ethanolic extract from *A. sativum* (500 mg/Kg)

**Table 1:** Qualitative results of phytochemical analysis of ethanolic extract of *Allium sativum*

**Phytochemical**	Extract
**Tannins**	+
**Phlobatannins**	-
**Cardiac glycosides**	+
**Flavonoids**	+
**Terpenoids**	+
**Phenolics**	+
**Reducing sugars**	-
**Steroids**	+
**Alkaloids**	+
**Anthraquinones**	+
**Saponins**	+

KEY: (+) = Present; (-) = Absent

Key: Group PC (Positive control): Control experiment fed with distilled water and food only

Group NC (Negative control): NDEA-induced rats only

Group A: NDEA-induced rats + ethanolic extract from *A. sativum* (50 mg/Kg)

Group B: NDEA-induced rats + ethanolic extract from *A. sativum* (100 mg/Kg)

Group C: NDEA-induced rats + ethanolic extract from *A. sativum* (200 mg/Kg)

Group D: NDEA-induced rats + ethanolic extract from *A. sativum* (300 mg/Kg)

Group E: NDEA-induced rats + ethanolic extract from *A. sativum* (400 mg/Kg)

Group F: NDEA-induced rats + ethanolic extract from *A. sativum* (500mg/Kg)

**Table 2:** Liver function test of the experimental organism

PARAMETER	**GRP A**	**GRP B**	**GRP C**	**GRP D**	**GRP E**	**GRP F**	**GRP NC**	**GRP PC**
AST (U/I)	35.67±8.50	28.00±5.00	26.33±4.16	28.00±2.65	28.67±2.08	26.33±3.51	15.00±3.08	14.00±1.00
ALT (U/I)	39.33±9.87	30.00±4.36	30.33±4.93	28.33±1.53	31.67±4.62	29.00±4.58	18.33±5.95	12.33±0.58
ALP (U/I)	295.00±3.61^d^	262.67±9.50^d^	247.33±16.92^d^	286.33±10.50^d^	261.00±15.13^d^	245.33±13.5^d^	190.67±65.13^d^	28.67±2.52
BILIRUBIN (g/dl)	2.40±0.46	0.90±0.10	1.30±0.69	2.17±0.45	0.87±0.15	1.37±0.64	0.57±0.49	0.30±0.10

Key: Superscript (d) means significance at *P*<0.001. Values in the table are expressed as mean and SD from triplicate data

GRP = group

Alanine aminotransferase (ALT), Aspartate aminotransferase (AST), Alkaline phosphatase (ALP)

Group PC (Positive Control): Control experiment fed with distilled water and food only

Group NC (Negative Control): NDEA-induced rats only

Group A: NDEA-induced rats + ethanolic extract from *Allium sativum* (50 mg/Kg)

Group B: NDEA-induced rats + ethanolic extract from *Allium sativum* (100 mg/Kg)

Group C: NDEA-induced rats + ethanolic extract from *Allium sativum* (200 mg/Kg)

Group D: NDEA-induced rats + ethanolic extract from *Allium sativum* (300 mg/Kg)

Group E: NDEA-induced rats + ethanolic extract from *Allium sativum* (400 mg/Kg)

Group F: NDEA-induced rats + ethanolic extract from *Allium sativum* (500 mg/Kg)

**Table 3:** Hematological profile of rats treated with varying concentrations of ethanolic extract of *Allium sativum* on NDEA-induced hepatocellular carcinoma. Triplicate values of the parameters are represented as mean and SD. Statistical significance at * = *P*<0.05, ** = *P*<0.01

A	B	C	D	E	F	Negative control	Positive control	Hematology profile
**18.30±8.28**	12.73±8.01	13.70±4.26	12.55±3.55*	21.17±6.37	14.97±6.98	35.77±2.36	22.53±4.82	**WBC**
**11.10±7.21**	5.53±3.70	5.67±1.70	5.80±1.00	9.10±2.87	5.33±2.02	26.63±4.12	9.50±1.65	**Lymph#**
**2.03±0.51**	1.93±1.40	1.90±0.80	1.60±0.60	2.67±1.07	1.83±0.45	3.03±2.73	3.40±0.46	**Mid#**
**5.17±1.02**	5.27±2.97	6.13±3.44	5.15±1.95	9.40±2.46	7.80±4.69	6.10±1.51	9.60±2.88	**Gran#**
**56.97±11.86**	42.73±2.42	42.53±10.49	47.40±5.10	42.90±1.11	36.73±6.22	49.60±4.96	42.43±1.92	**Lymph%**
**12.03±4.30**	14.50±1.87	14.13±4.68	12.55±1.15	12.23±1.60	12.97±2.66	5.70±4.94	15.57±2.60	**Mid%**
**31.00±9.02**	42.77±4.07	43.87±4.14	40.05±3.95	44.87±2.20	50.30±8.62	11.37±9.84	42.00±4.50	**Gran%**
**16.90±1.83**	16.37±4.41	17.03±2.05	13.80±1.90	23.57±5.59	15.93±3.78	13.57±2.19	19.77±8.42	**HGB**
**8.73±0.84**	7.16±0.46	8.87±0.23	8.01±0.49	7.62±1.27	7.63±0.79	5.69±4.95	7.71±0.38	**RBC**
15.97±7.66	11.23±1.46	40.63±8.53	24.25±4.25	0.00±0.00	6.53±3.17	16.13±2.94	14.40±4.94	**HCT**
52.80±1.21	53.03±2.06	52.93±2.85	58.20±6.30	50.97±6.42	54.00±4.21	35.93±31.12	53.87±2.72	**MCV**
18.53±0.67	17.90±1.25	18.30±0.52	16.60±0.80	17.77±0.64	18.80±1.25	12.77±11.06	17.83±0.35	**MCH**
33.70±0.96	33.37±0.91	34.50±1.10	29.20±4.70	33.90±1.41	36.93±2.45	23.80±2.62	33.83±1.82	**MCHC**
14.60±1.73	16.13±1.25	16.13±0.38	16.70±1.90	14.60±1.74	15.07±1.03*	9.83±8.74	14.47±1.12	**RDW-CV**
26.80±1.73	29.57±1.42	28.43±2.10	33.15±6.95	25.90±2.12	26.90±1.35	17.77±5.86	26.97±3.62	**RDW-SD**
920.33±18.13	916.33±29.73	770.00±32.3	2039.00±61.00	861.33±25.8	951.33±38.2	771.33±87.59	1085.67±63.96	**PLT**
7.40±0.66	6.90±0.40	7.13±0.47	7.75±0.45	7.03±0.23	7.50±0.70	5.60±1.85	7.57±0.55	**MPV**
16.00±0.70	16.47±2.11	16.03±0.81	15.65±0.25	15.30±0.17	16.30±0.82	11.43±2.90	15.83±0.84	**PDW**
0.39±0.34	0.35±0.33	0.29±0.29	0.00±0.00	0.51±0.11	0.37±0.35	0.00±0.00	0.00±0.00	**PCT**

**Plate 8:**
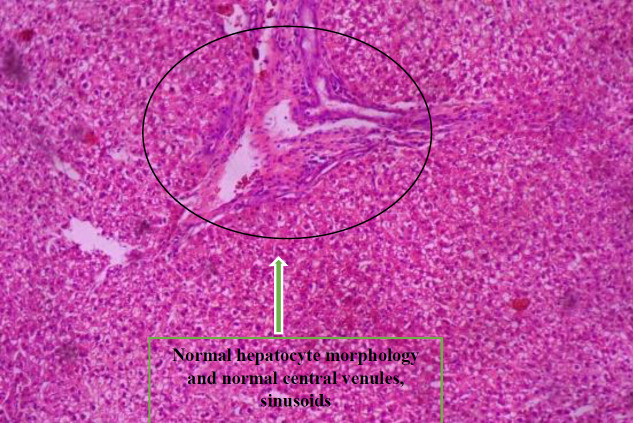
Histologic sections of liver tissue of group G showing normal hepatocyte morphology, normal central venules, and sinusoids. Group PC (Positive Control): Control experiment fed with distilled water and food only

Key: WBC: white blood count; RBC: Red blood count; lymph#: lymphocyte count; Mid: mid-range absolute count; Gran#: granulocyte count; Lymph%: lymphocyte percentage Mid%: mid-range percentage count; Gran%: Granulocyte percentage; HGB: Hemoglobin; HCT: Hematocrit; MCV: Mean Corpuscular Volume; MCH: Mean Corpuscular Hemoglobin; MCHC: Mean Corpuscular Hemoglobin Concentration; RDW-CV: Red Cell Distribution Width; PLT: Platelet Counts; MPV: Mean Platelet Volume; PDW: Platelet Distribution Width; PCT: Procalcitonin

**Plate 9:**
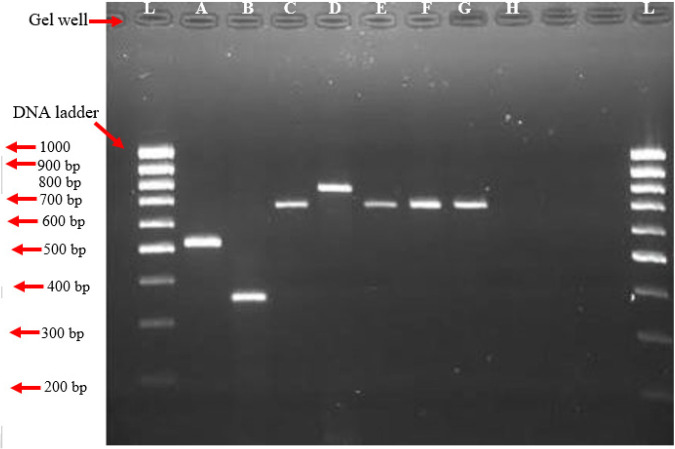
Amplification of *TP53* gene from DNA isolated from blood tissue by gel electrophoresis. Legend: L: Molecular marker; A: M0500; B: M0350; C: M0700; D: M0800; E-G: M0700

## Discussion

Liver cancer remains one of the most common human cancers with a high mortality rate. Conventional therapies for HCC remain ineffective, due to the heterogeneity of HCC with regard to both etiology and mutation spectrum, as well as its chemotherapy-resistant nature (3, 20). HCC is the most common type which accounts for about 80% of primary liver cancers and is a leading cause of cancer-related deaths worldwide (21–24). There is a striking variation in HCC incidence rates across geographic regions and at the global level, each year over 800,000 people are diagnosed with liver cancer (25, 26). The current therapeutic option in cancer management requires the use of herbal remedies and medicinal plants, since the majority of anticancer drugs are known to be costly followed by unwanted side effects (27). 

In this present study, the anti-cancer property of *A. sativum* was investigated in albino rats induced with hepatic cancer using NDEA. Results obtained from the phytochemical analysis of ethanolic extract of *A. sativum* were used to reveal the presence of saponin, alkaloid, anthraquinones, terpenoids, tannins, flavonoids, steroid, phenol, and glycoside, while reducing sugar, and phlobatannins was absent (Table 1). According to Ali and Ibrahim (11), garlic is rich in organosulfur, tannins, glycosides, and other phytochemicals that take part in biological effects. The anticancer potential and activity are presumably the most studied among the various beneficial pharmacological effects of garlic. Consumption of garlic provides strong defense against cancer occurrence. A few active metabolites of garlic (*A. sativum)* play important roles in killing of cancerous cells, taking into account the multi-targeted actions and absence of considerable toxicity (28).

The effect of different concentrations of garlic extract on the weight of the albino rats that were administered with NDEA is shown in Figure 1. This finding suggested that garlic extract neutralized the negative body weight effects of NDEA carcinogenic properties observed in albino rats treated with a lesser concentration of garlic extract and the negative control group. The body weight analysis was further supported with findings from the mortality rate analysis which showed a remarkable decrease in the survival probabilities of groups of albino rats treated with lesser concentrations of *A. sativum* extract (Figure 2). This result agrees with the anti-carcinogenic properties of phytochemicals present in the garlic extract used in this study just as reported by Zhang *et al*. (29). Garlic components also play important roles in the progression of carcinogenesis in invasive and metastatic cancerous cells. It is widely recognized that extraction boosts the potency and bioavailability of various bioactive compounds of herbal plants, including garlic, and decreases harsh and toxic characteristics (29, 30).

NDEA primarily induces the formation of hepatocellular carcinoma in rats and mice and is also used as a model hepatic carcinogen in experimental studies of carcinogenesis and chemoprevention (31). In this study, serum enzymes were evaluated to determine the anticancer potential of *A. sativum* extract on NDEA-induced hepatocellular carcinoma in rats (Table 2). The ethanolic extract of *A. sativum* caused a significant (*P<*0.05) increase in serum Alanine aminotransferase (ALT), Aspartate aminotransferase (AST), Alkaline phosphatase (ALP), and an increase in the level of serum total bilirubin was observed in the treatment groups when compared with the control group (NDEA-induced rats only) (Table 2); which suggests that *A. sativum* could effectively impede NDEA-induced hepatocellular damage. Serum Alanine aminotransferase (ALT) and Aspartate aminotransferase (AST) are recognized as excellent biomarkers of hepatocellular injury. They participate in gluconeogenesis by catalyzing the transfer of amino groups from alanine or aspartic acid to ketoglutaric acid to produce pyruvic acid and oxaloacetic acid respectively (20).

Histological examination reveals the photomicrographs of histological sections of liver tissues in treatment and control groups starting from group A to H (Plates 1–8). These results showed hepatocytes surrounded by broad fibrous band and degeneration, necrotic hepatocytes, and liver cirrhosis with malignant transformation in NDEA-induced untreated group while the groups treated with ethanolic extract of *A. sativum* showed some level of distortion in the liver architecture and liver sections with normal liver morphology, and venules with normal hepatocytes. In a further corroboration study (32), histological assessment revealed hepatocellular carcinomas and dysplastic nodules in the liver of NDEA-treated animals characterized with cirrhosis, dilatation of bile ducts, and ballooning degeneration.

Results from this present study also investigated the hematological parameters of the NDEA-induced rats across all groups treated with ethanolic extract of *A. sativum* (Table 3). The blood indices (lymphocytes, white blood cells, red blood cells, platelets, and their differentials) serve as indicators of physiological and pathological status of the body and significant changes imply that the administered chemical is either protective or toxic to the hemopoietic tissue (33). The results showed that WBC and lymphocytes in experimental rats induced with NDEA only (PC) decreased significantly compared with the normal control (NC), while packed cell volume (PCV), hemoglobin, red blood cells, hemoglobin, Platelet counts (PLT), and Mean Platelet Volume (MPV) increased significantly compared with the normal control (NC). There was no significant difference in WBC count in the treatment groups except group D compared with the control. The major functions of WBCs and their differential are to provide immunity and defend the body against invasion by pathogens or toxins. Therefore, the non-significant difference in WBC count and its differentials between the treatment and control groups suggested that the administered doses did not interfere with differentiation of hemopoietic stem cells into these parameters. The significant effect on RBC differentials indicated that the extract affected the process of erythropoiesis probably by the phytochemicals interfering with the secretion and/or activity of erythropoietin (33–36). There was a significant reduction and absence of procalcitonin (PCT) in both treatment and control groups, respectively. Diminished levels of procalcitonin could probably be due to the presence of toxic phytochemicals that interfere with the functioning of thrombopoietin or cause inflammation of the bowel (37). 

Results from this study further revealed that the difference in the band pattern of the tumor suppressor p53 (*TP53)* gene was involved in the advancement of the hepatocellular carcinoma observed in albino rats. This was evident by the presence of the amplified gene during PCR (Plate 9). DNA bands were observed in groups A to G induced with NDEA and treated with varying concentrations of ethanolic extract of *A. sativum* with molecular weights of (523, 358, 691, 780, 691, and 691) base pairs, respectively while the positive control (group H) showed no band and the Negative control (group G) had 691 base pairs. This finding suggests that DNA damage in hepatocellular carcinoma was caused by induction of NDEA thereby activating and elevating the TP53 gene. Tumor suppressor p53 levels under normal conditions are maintained at a low state by virtue of the extremely short-half life of the polypeptide and normally exists largely in an inactive state that is relatively inefficient at binding to DNA and activating transcription. Activation of p53 in response to DNA damage is associated with a rapid increase in its levels and with an increased ability of p53 to bind DNA and mediate transcriptional activation. This then leads to the activation of a number of genes whose products trigger cell-cycle arrest, apoptosis, or DNA repair. Several forms of DNA damage have been shown to activate p53, including those generated by chemicals such as methyl methane sulfonate (MMS), ionizing radiation (IR), ultraviolet light (UV), and radio-mimetic drugs (38–40). This in turn infers that the mutation of the TP53 gene is consequential to the emergence of hepatocellular carcinoma just as stated by Villanueva and Hoshida (41) that there is a strong association between TP53 mutations and HCC*. *TP53 mutation is one of the most common genetic changes in HCC. It is of great clinical significance to tailor the specialized prognostication approach and to explore more therapeutic options for TP53-mutant HCCs (37).

## Conclusion

Liver cancer remains a global health challenge. HCC is the most common form of liver cancer and accounts for ~90% of cases. Most of the current cancer chemotherapeutics and surgery, chemotherapy, radiotherapy, hormonal therapy are associated with harsh and undesirable side effects, including toxicity and chemoresistance, driving the need for safer and more effective treatment alternative The phytochemical analysis of ethanolic extract of *A. sativum* revealed the presence of the main groups of bioactive chemical compounds such as tannins, alkaloids, terpenoids, flavonoids, steroid, phenol, anthraquinones, saponin, tannin, and glycosides. The present study confirms the anti-cancer properties of ethanolic extract of *A. sativum* on NDEA-induced hepatocellular carcinoma and recommends its usage as an alternative medication for the treatment of liver cancer.

## Authors’ Contributions

OG and MJ Conceived the project and defined the research methodology; CJ, OO, OS, and OM Performed the experiments; OG, MJ, BJ, and AA Wrote the paper.

## Funding

None.

## Availability of Data Set and Materials

The data sets used and/or analyzed during this study are available from the corresponding author upon reasonable request.

## Ethical Approval

The work was carried out in the animal house (Botanical garden) University of Lagos in accordance with the Code of Ethics of the World Medical Association for Animal Experiments. Approval was obtained from University of Lagos Ethics Committee for experiments with whole animals.

## Conflicts of Interest

The authors declare that they have no competing interests.
